# Persistent truncus arteriosus: one trunk, one defect - an image telling the story

**DOI:** 10.11604/pamj.2025.51.98.48406

**Published:** 2025-08-19

**Authors:** Zaynab Laoufi, Nadia Fellat

**Affiliations:** 1 Cardiology A Department, Ibn Sina University Hospital Center, Rabat, Morocco

**Keywords:** Echocardiography, persistent truncus arteriosus, ventricular septal defect

## Image in medicine

Persistent truncus arteriosus is a rare congenital heart defect characterized by a single arterial trunk arising from the heart and supplying the systemic, pulmonary and coronary circulations. It is frequently associated with a large ventricular septal defect (VSD), leading to mixing of oxygenated and deoxygenated blood. This malformation results from the failure of the embryonic truncus arteriosus to divide into the aorta and pulmonary artery during fetal development. We present the case of a 19-year-old woman with a history of persistent truncus arteriosus diagnosed during infancy. Surgical correction was proposed but declined due to a lack of resources. She was admitted for evaluation of syncope. Clinical examination revealed a diastolic murmur at the left sternal border. Electrocardiogram (ECG) showed frequent premature ventricular contractions (PVCs) in bigeminy. Transthoracic echocardiography revealed a large ventricular septal defect (VSD) underlying a single arterial trunk, consistent with persistent truncus arteriosus, with a well-functioning truncal valve exhibiting mild regurgitation. Twenty-four-hour Holter monitoring confirmed frequent PVCs, including short runs of non-sustained ventricular tachycardia. The patient was treated with a beta-blocker and spironolactone, showing fewer PVCs at one-month ECG follow-up, and was referred for surgical management.

**Figure 1:**
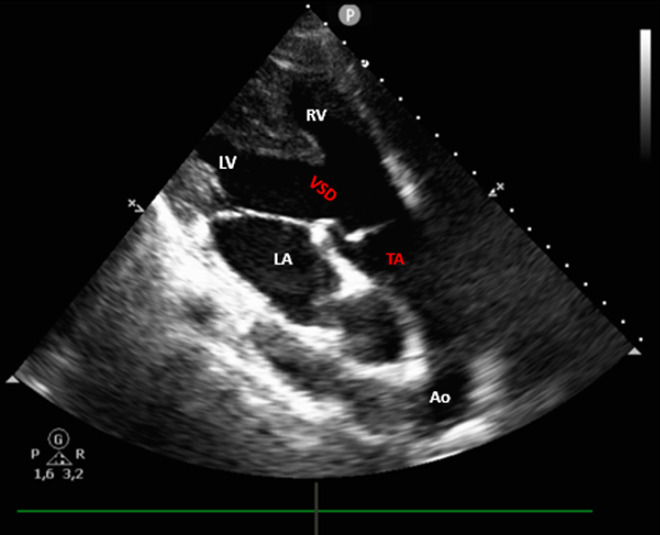
parasternal long-axis transthoracic echocardiographic view demonstrating a single arterial trunk (TA) arising above a large ventricular septal defect, in continuity with a competent truncal valve; the aortic arch is clearly visible, illustrating the branching of the great vessels (LA: left atrium; LV: left ventricle; RV: right ventricle; TA: truncus arteriosus; VSD: ventricular septal defect; Ao: aorta)

